# Lateral Differences in Cutting Abilities in Team Handball Athletes

**DOI:** 10.5114/jhk/200617

**Published:** 2025-07-21

**Authors:** Frowin Fasold, Patrick Engel, Stefanie Klatt

**Affiliations:** 1 Section Cognition in Team Sports, German Sport University, Cologne, Germany.

**Keywords:** change of direction, agility, team sports, performance, movement speed

## Abstract

Change of direction movements are highly relevant for performance in team sports. While bilateral movement patterns appear advantageous, research indicates unilateral dominance in cutting manoeuvres among handball athletes. This means, cutting manoeuvres in the direction of the throwing arm are executed faster than those against the direction of the throwing arm. The aim of this study was to evaluate whether this unilateral dominance stemmed from differences in physical capabilities. We predicted that the specific speed differences in cutting could be explained by lateral power differences. Thirty-two handball athletes completed lateral jump tests and a handball-specific change of direction test. Movement speed during the test was determined via video analysis. Participants showed significantly higher movement speed and significantly higher jump distances in the direction of the throwing arm. However, a significant interaction for both variables indicated that differences in the jump width could not fully explain the differences in movement speed. This likely indicates that the reasons of unilateral differences in cutting manoeuvres are not only based on physical capabilities. Coordinative-technical aspects or psychological variables should be investigated in further research as basis for an evidence-based training concept in educating the bilateral unpredictable abilities in handball players.

## Introduction

Performance in sports is inherently multifactorial and multidimensional. According to Bangsbo’s (2015) holistic model of performance, technique, tactics, physiology, and psychology are identified as potential determinants of performance. Such models serve to elucidate performance levels for the development of holistic training programs and the identification of specific performance variables. However, the complexity inherent in team sports complicates precise identification (Gómez-López et al., 2024). When dissecting performance in a game into its underlying actions, determining whether success or failure primarily stems from technical or tactical skills, physical capabilities, or social and psychological abilities becomes challenging. At the individual level, changes of direction and agility movements appear to impact game performance (Forster et al., 2022; Nimphius et al., 2018; Wagner et al., 2014). In team handball, ones of such specific agility movements are offensive cutting manoeuvres with the ball (Bagehorn et al., 2024). Fasold et al. (2022) demonstrated a lateral asymmetry in these specific cutting manoeuvres, wherein athletes exhibited higher change of direction speed in the direction of their throwing arm. When this result is considered in conjunction with the performance determinants of Bangsbo (2015), it is questionable whether technical-coordinative skills, athleticism (physiological) or motivational (psychological) factors could be taken into account to explain these differences.

Lateral power and strength disparities in handball athletes have been shown in several investigations using, amongst others, isokinetic strength tests (Horn et al., 2019), standardized jump tests (Cadens et al., 2023), and change of direction tests (Madruga-Parera et al., 2020). However, it remains unclear whether such athletically determined disparities are responsible for the asymmetries in movement speed observed during specific cutting manoeuvres, as reported by Fasold et al. (2022).

To evaluate this relationship, cutting manoeuvres can be contextualized within the concept of *agility*. Haff and Tripplet (2016) defined agility as the ability to change direction or alter one’s speed and the type of movement in response to a sport-specific stimulus. In contrast, changes executed under pre-planned conditions (without responding to a stimulus) are classified simply as *changes of direction*. According to Young et al. (2021), particularly in team sports, distinguishing between agility and change of direction is crucial, as the tactical nature of such sport games requires that change of direction movements incorporate cognitive factors such as perception, anticipation and decision making. This implies that in sports, almost every change of direction entails a movement governed by the principles of agility. Young and Farrow (2006) conceptualized agility as a multifaceted ability. Their model offers a nuanced perspective on this complex skill by delineating cognitive factors including anticipation, pattern recognition, and visual scanning, along with motoric factors such as sprinting speed, technique, and muscle qualities (Young and Farrow, 2006). Based on this model, we may assume that the motor part (in this case, change of direction speed) could be seen as the physical capacity in agility actions. While both cognitive and physical capacities are examined in research, and frameworks have been proposed categorizing athletes based on variables related to speed in both thought and movement (Gabbett and Sheppard, 2013), it remains unclear which aspect holds greater significance in explaining performance. From a holistic perspective, we can anticipate a strong correlation between cognitive abilities and the physical capacity in executing specific agility actions. However, this study would narrow its focus to the motor component by simplifying complex agility actions to the ability to change the direction.

Nevertheless, agility abilities and the change of direction skills are sport-specific (Loturco et al., 2022; Salaj and Markovic, 2011) and in team handball, in particular stop-and-go movements are required (Sekulic et al., 2014; Spasic et al., 2015). Side-cutting manoeuvres with and without possession of the ball are specific actions and require stop-and-go change of direction movements of athletes (Michalsik, 2018). Fasold et al. (2022) investigated the movement speed in such a cutting maneuver with the ball and found a specific imbalance between two movement vectors. Particularly, in the direction of the throwing arm (right-handers to the right, left-handers to the left), players performed faster cutting manoeuvres than against their throwing arm. These effects remained stable under planned change of direction conditions and under agility conditions (in response to a visual stimuli). With a view on the unilateral dominance in handball where a lot of game actions are performed unilaterally (Janicijevic et al., 2023), these effects seem plausible, although are surprising, because bilateral edification during change of direction is a crucial factor for athletic performance (Arboix-Alio et al., 2020). Bilaterality makes athletes more unpredictable, which could be seen as an advantage in multidirectional sports actions (Arboix-Alio et al., 2021; Dos' Santos et al., 2019). Meanwhile, the reasons of the vector specific imbalances in change of direction speed shown by Fasold et al. (2022) are still unclear. If we consider that physical capabilities of athletes are responsible, they should be explained by general imbalances in strength or power.

Based on the limited body of research on specific change of direction movements in handball, the causes of the lateral speed imbalances are difficult to predict. However, the relationship between change of direction abilities, speed, and physical capabilities (e.g., power, strength) has been examined in previous studies. Horníková and Zemková (2021) demonstrated a strong association between general change of direction speed, sprint speed, muscle strength, and jumping power. The strength of this correlation varies depending on the type of directional change and the variables analysed (e.g., height, power demands, velocity). Delaney et al. (2015) found no lateral differences in speed and no disparities in lateral power output (as measured by a single leg lateral jump) in change of direction tests. Although their study focused on professional rugby players, they highlighted the contribution of lateral jumping power to change of direction performance in a sport with similarities to team handball.

Building upon the established relationship between power capabilities (e.g., jumping) and performance in change of direction movements (Delaney et al., 2015; Forster et al., 2022; Horníková and Zemková, 2021), we hypothesized that lateral jumping power could explain the lateral speed differences observed in handball-specific cutting manoeuvres (as reported by Fasold et al., 2022). Specifically, we anticipated that higher movement speed in the direction of the athletes’ throwing arm would correlate with superior jumping performance in that direction compared to the opposite direction (against the throwing arm).

Given the typical lateral dominance observed in handball athletes, where right-handers predominantly use their left leg for jumping (e.g., during jump shots) and left-handed players use their right leg (Özkamci et al., 2022), we expected a cross-lateral power advantage. This would imply that the leg opposite to the throwing arm would generate more power (e.g., speed, jump distance), a finding supported by previous studies, even if the effects observed were small (Arboix-Alio et al., 2021; Cadens et al., 2023).

## Methods

### Participants

A mean effect size was calculated based on the directional effect described by Fasold et al. (2022) (*f* = 0.93). Using G*Power (Faul et al., 2009), it was calculated that a sample size of 32 participants would achieve a power of 0.99.

Overall, 17 males and 15 females participated voluntarily in this study (mean age 22.29 years, *SD* = 2.05 years). All participants reported that they had played minimum one year in the 5^th^ league in Germany, and were still active in training and playing handball. Participants at this level, though not professionals, were expected to have mastered handball basics (e.g., performing cutting maneuvers with the ball). Goalkeepers and athletes who were injured (injuries of lower extremities) in the past 18 months, were excluded from the study as this could impact the results. Three of the male participants were left-handed. The study was approved by the Ethics Committee of the German Sport University, Cologne, Germany (protocol code: 088/2021; approval date: 08 June 2021) and was carried out according to the declaration of Helsinki of 1975 and its later revisions. Written informed consent was obtained from each participant before testing.

### Design

We investigated the effect of the within-subject factor *direction* (in the direction of the throwing arm vs. against the direction of the throwing arm) on the dependent variables *speed* in a handball specific change of direction (cutting manoeuvre) and *distance* in a lateral jump test. As a covariate, the between subject factor of *gender* was integrated into the study design to ensure consistency of the results across male and female participants with regard to the factor of direction.

### Procedure

Participants engaged in an individual warm-up session. Following the warm-up, all players completed three counter-movement jumps and three broad jumps in the same sequence as a standardized activation procedure (distances were measured, but not used for analysis).

Since lower limb power is often assessed through jumping tests (Delaney et al., 2015; Meylan et al., 2009) and change of direction performance is strongly linked to jumping capabilities (Castillo-Rodríguez et al., 2012), we chose lateral jumps to measure specific power abilities. For lateral jumps, participants were positioned with the medial edge of the jumping foot on the starting line, while the non-jumping foot had to be airborne. It was allowed to use a free arm and leg insert. The landing had to be on both feet at the same time. The lateral jumps were executed according to the protocol outlined by Lockie et al. (2015) and measured using a measuring tape. Each participant completed three jumps with each leg. In the event of a failed attempt, the jump was allowed to be repeated. Jumping, in various forms, is a standard component of (physical) training in handball; therefore, we expected that all participants would be familiar with the demands of the lateral jump test.

**Figure 1:**
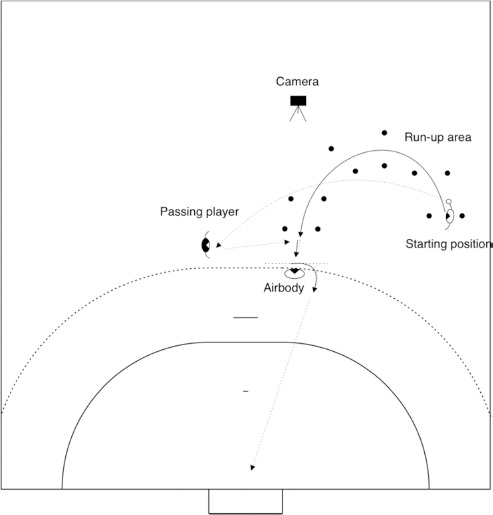
Procedure and materials used in the change of direction test. Note: the procedure was mirrored on the left side of the handball field, if participants were left-handed

For testing the change of direction speed, the setup and the procedure of Fasold et al. (2022) was partly replicated in this study. A description of the setup is shown in Figure 1. Plates marked the run-up. An artificial defender via usage of an air body was used. Participants could choose the ball size (ball size two or three). They were asked to choose a ball they felt most comfortable with. A video camera (iPad 5^th^ generation, Coach’s Eye application TechSmith Corp. Version 6.6.0, recording rate of 240 Hz), was used to record the change of direction action.

Tape strips were placed on the floor at 10 cm apart, parallel to the goal line with 9-m distance. This line was used to measure the change of direction distance in front of the air body. In this study, the strips were large enough to be visible on the ground in the video, but small enough not to influence the participant’s movements.

Participants started 1.85 m far from the side-line and 11.20 m from the goal line in the starting position (Figure 1). After that, they passed the ball to the passing player who was positioned at the height of the opposite goal post, 10 m from the goal line. They ran in a 1.35-m wide corridor marked by plates (a curved run-up). The run-up was designed like in several game actions. There were two last plates in the run-up area, and they were located 7.80 m from the side-line (in the centre between the two plates) and 10.70 m from the goal line.

Participants received the ball in the receiving zone, which was located 8 m from the side line and 9 m from the goal line. The passing player passed the ball to them. The air body was used to simulate a defender. After participants received the ball, they executed a 1:1 action (change of direction, cutting manoeuvre) without contact, against the air body. This action was followed by a throw to the goal with a jump shot (Figure 1). Every participant repeated these actions six times under two conditions: 1) in the direction of the throwing arm, and b) against the direction of the throwing arm. The sequence of the conditions was balanced by the number of participants. Participants had 40 s of rest between each of the six trials. When participants were left-handed (*n* = 3), the procedure was mirrored on the other side of the field.

### Data Preparation

The study procedure was led and the date preparation was conducted by an experienced sport scientist (age 25) with five years of handball specific coaching experience (B-level license).

As described above, the lateral jump distance (m) was assessed using a measuring tape and subsequently recorded.

To measure movement speed, we adopted the procedure outlined by Fasold et al. (2022). The video recordings were utilized to measure the distance (m) from the initial foot contact to the landing of the foot after the lateral movement (as indicated by the line of the tape stripes). The time taken for this lateral movement (s) was calculated based on the number of video frames (between the initial contact and the landing). Movement speed (m/s) was then determined by dividing the space gain by the time. Further details of the procedure can be found in Fasold et al. (2022).

Analyzing performance metrics such as seconds and distances through subjective observations may introduce potential inaccuracies, but similar methods have demonstrated reliability in measuring physical performance (Gallardo-Fuentes et al., 2016). Additionally, conducting multiple trials and using mean values can help mitigate subjective biases.

### Statistical Analysis

In a 2 x 2 design, a single factor MANCOVA with repeated measures was used to evaluate the effect of the within factor *direction* (in the direction vs. against the direction of the throwing arm) and the between cofactor *gender* (female vs. male) on the dependent variables *movement speed* (in the cutting manoeuvre) and *lateral jump distance*. For the *movement speed*, the mean values of all six attempts (per direction) were calculated to make the variable robust against failures in the measurement. Since the jumping power was defined as the physical capacity of the change of direction movement (see above), and the measurement could be done quite precise, the best attempt was used for the variable *lateral jump distance*. As an effect size η^2^_partial_ was calculated, by assuming values of < 0.009 as small, 0.05 as moderate and > 0.13 as large (Richardson, 2011).

Based on the assumptions and previous research (Fasold et al., 2022), we expected to find an effect of the direction factor (and the covariate gender) on the dependent variables. Furthermore, considering that the lateral movement speed in the cutting manoeuvre is linked to lateral jumping power, there should be no significant interaction in the dependent variables. This means, the direction differences in the cutting speed should be strongly related to direction differences in the lateral jumping distance.

## Results

All assumptions for the MANCOVA analysis were met, as confirmed by the Shapiro- Wilk test, which indicated a normal distribution under all conditions (Table 1). The covariate *gender* revealed a significant main effect, with male athletes performing the cutting manoeuvres faster (*M* = 4.60 m/s, *SD* = 0.78, *CI* 95% [3.88, 5.00]) and jumping farther (*M* = 1.91 m, *SD* = 0.14, *CI* 95% [1.83, 1.98]) compared to female participants (speed *M* = 4.13 m/s, *SD* = 0.71, *CI* 95% [3.73, 4.52]; distance *M* = 1.63 m, *SD* = 0.10, *CI* 95% [1.57, 1.68]), *F* (1, 30) = 4.45, *p* = 0.002, η^2^_partial_ = 0.27). This effect was stable under all conditions, as evidenced by the lack of significant interactions with the other variables (gender*speed*distance, *F* (1, 30) = 1.47, *p* = 0.23, η^2^_partial_ = 0.04; gender*speed, *F* (1, 30) = 0.91, *p* = 0.34, η^2^_partial_ = 0.03; gender*distance, *F* (1, 30) = 1.00, *p* = 0.32, η^2^_partial_ = 0.03).

**Table 1:** Descriptive data for the dependent variables movement speed (m/s) and lateral jump distance (m) under both directional conditions.

	Movement direction						
	Direction of throwing arm				Against direction of throwing arm		
	Speed in m/s		Distance in m		Speed in m/s		Distance in m
*M*	4.90*		1.81†		3.86*		1.74†
*SD*	0.64		0.10		0.78		0.10
*CI* 95%	4.66, 5.13		1.73, 1.88		3.53, 4.19		1.68, 1.80
Shapiro-Wilk *p* (male, female)	0.22, 0.14		0.58, 0.97		0.57, 0.14		0.97, 0.07

Note: * indicates the significant main effect of the factor direction on the variable speed; † Indicates the significant main effect of the factor direction on the variable distance; M represents the mean, SD the standard deviation and CI 95% the confidence interval. The Shapiro-Wilk p-values demonstrate the normal distribution of the data under all experimental conditions (separated by the covariate gender)

The factor *direction* showed a main effect on speed (*F* (1, 30) = 16.43, *p* < 0.001, η^2^_partial_ = 0.63). Movements in the direction of the throwing arm were conducted faster than movements against the direction of the throwing arm. A similar directional effect was observed for jump distance (*F* (1, 30) = 9.05, *p* = 0.005, η^2^_partial_ = 0.23). The jump distance was greater in the direction of the throwing arm than against it (Table 1).

The interaction between speed and distance was also significant (*F* (1, 30) = 8.00, *p* = 0.008, η^2^_partial_ = 0.21), indicating that although both variables exhibited a similar directional effect, the differences in speed were significantly greater than those in jump distance (Table 1). This suggests that the differences in the lateral jump distance do not fully account for the differences in speed.

## Discussion

The aim of this study was to assess whether lateral disparities in change of direction speed during specific cutting actions in team handball athletes could be attributed to lateral (jumping) power discrepancies. First, the results replicated the findings of Fasold et al. (2022), demonstrating a lateral speed disparity in specific cutting maneuvers with the ball. Irrespective of the gender covariate, participants exhibited faster change of direction movements when cutting toward their throwing arm, with an increase of 22% compared to the opposite direction. Second, our findings revealed that participants displayed superior lateral jumping performance in the direction of their throwing arm, with an increase of 4%. However, the significant interaction between change of direction speed and lateral jumping distance in the directional condition suggests that the observed improvement in jumping distance alone cannot fully explain the enhancements in change of direction performance toward the throwing arm side. This implies that participants performed better in both dependent variables (change of direction speed and lateral jumping distance) when moving in the direction of their throwing arm, but these improvements were independent of each other. The extent of improvement in physical capabilities (lateral jumping distance) could not account for the observed performance enhancement in the specific cutting maneuver (speed), contradicting our initial assumption.

Additionally, the observed lateral power differences were small, and following the results of Carrasco-Fernández et al. (2023), we did not expect that this differences would be based on disbalances in mass (they found no differences in muscle and fat mass between both legs). Nevertheless lateral strength and power imbalances, as we found in our study, have been documented in previous investigations involving handball athletes (Cadens et al., 2023; Horn et al., 2019; Madruga-Parera et al., 2020). Differences exceeding 15% are associated with a predisposition for injury (Xaverova et al., 2015), but given the results of our study, the observed imbalance in lateral jumping power might be considered non-critical in terms of injury predisposition.

However, considering that unpredictable bilateral skills provide a competitive advantage (Arboix-Alio et al., 2021) to overpower the opponent, training and exercises should be developed to reduce the lateral speed difference in handball athletes, by increasing their movement speed against the direction of the throwing arm. As we defined that the motoric part of agility (Young and Farrow, 2006) could be seen as the physical capacity of agility skills, we expect that physical training could be used to reduce the speed differences in specific lateral cutting movements in handball. Forster et al. (2022) showed in their review that improvements in agility performances could be induced by various methods of physical training. Sprinting may develop especially the linear acceleration phases; unilateral resistance training may promote increased strength to overcome the imposed forces during the deceleration and acceleration (in change of direction). Multidirectional plyometrics enhance the capabilities of the stretch-shortening cycle across various force vectors. A combination of at least two of these methods could potentially result in a simultaneous improvement of the individual performance variables in agility movements (Forster et al., 2022). Additionally, based on the findings of Pori et al. (2023), unilateral strength and power training is particularly effective in developing specific athletic skills. This means that performing counter movement jumps with one limb may be more beneficial than their execution with both limbs. According to these shown evidence-based training effects in specific agility variables, we expect that physical training interventions could be used to reduce the observed differences in our study at the level of physical capacity. However, considering the results of our study, it appears that merely reducing differences in the physical aspect of lateral movement abilities may not be adequate to foster an unpredictable bilateral performance.

Considering the multifactorial nature of performance in team sports (Bangsbo, 2015), ongoing studies should further evaluate the technical aspects of cutting manoeuvres in handball. Sarvestan and colleagues (2022) evaluated kinematics of unspecific changes of direction using the dominant and non-dominant legs of basketball and football athletes. Although they did not find lateral performance differences, significant disparities in movement kinematics were observed. Based on this, we may expect that technical variables could contribute to the reduced movement speeds of handball athletes when moving in the direction against their throwing arm. Complementary, leg laterality effects among handball players are primarily assessed within non-specific paradigms (Madruga-Parera et al., 2020). Therefore, future research should investigate the impact of leg laterality (leg dominance) on lateral cutting actions in handball.

While our study focused on specific physical capacities and their influence on lateral movement speed, we acknowledge that performance in team sports is inherently multifactorial. Achieving balanced bilaterality can enhance unpredictability and serve as an tactical advantage (Arboix-Alio et al., 2021; Dos' Santos et al., 2019). However, our study aimed to isolate and assess the impact of lateral power disparities on movement speed, providing valuable insights into this specific aspect of handball performance. It is important to note that our findings do not negate the relevance of other factors, such as technical proficiency, psychological factors, overall game strategy or simple frequency effects, in shaping performance outcomes. Rather, they highlight the need for a comprehensive understanding of the various factors influencing performance in team sports. Therefore, future research should continue to explore the interplay between different determinants to develop a more holistic understanding of performance dynamics in handball and other team sports. For instance, simple frequency effects could be a promising area of exploration. Performing more actions in the direction of the throwing arm compared to the opposite side may lead to neuronal adaptations, potentially explaining the speed and power differences observed in our study. Furthermore, psychological factors may contribute to the lateral speed disparities. If athletes feel more comfortable moving in the direction of their throwing arm, their performance may improve, as heightened intrinsic expectancies have been shown to enhance motor performances (Wulf and Lewthwaite, 2016). Future research combining kinematic analyses with qualitative movement observations and evaluations of athletes’ motivation could provide deeper insights into the underlying causes of lateral differences in movement speed in handball. Such a comprehensive approach could inform the development of targeted training strategies designed to optimize athletes’ attacking performance in handball.

While our study encompassed a sample size of 32 participants, and a power analysis was conducted to ensure adequate statistical power, it is important to acknowledge the potential limitations inherent in our sample. Although certain results could be replicated (Fasold et al., 2022), future research involving larger sample sizes and participants from diverse performance levels could serve to enhance the validity of our findings and offer additional insights into the lateral differences in cutting abilities among team handball athletes. Although the handedness of participants (left-handed vs. right-handed) was not a primary focus of our study, we must acknowledge that left-handed individuals were underrepresented in our sample, making a statistical analysis based on this factor unfeasible. However, we suggest that future studies should specifically examine left-handed players, as they often practice under conditions designed for right-handed athletes due to their general underrepresentation. Based on this assumption, it could be argued that general laterality effects might be less pronounced in left-handed players. In addition to this assumption, position-specific differences should be considered. As Alanen et al. (2023) showed in soccer players, the frequency and execution of changes of direction varied depending on the playing position. Similarly, we expected in handball that playing position experience could influence the execution of cutting maneuvers.

## Conclusions

In conclusion, this study disproves the assumption that lateral differences in change of direction actions are significantly linked to the specific physical capacities of athletes’ agility (lateral jumping power). While participants in our study exhibited significant lateral differences in jumping power, these disparities could not fully account for the lateral speed differences in cutting manoeuvres. Before practical training implications can be recommended, ongoing research is necessary to clarify the reasons behind the lateral differences in such sport-specific agility actions.
